# Long COVID: Is there a kidney link?

**DOI:** 10.3389/fmed.2023.1138644

**Published:** 2023-04-03

**Authors:** Raymond E. Garrett, Carlos H. Palacio, David Bar-Or

**Affiliations:** ^1^Swedish Medical Center, Trauma Research Dept., Englewood, CO, United States; ^2^South Texas Health System, Trauma Research Dept., McAllen, TX, United States

**Keywords:** SARS-CoV-2 (COVID 19), kynurenine (KYN), creatine phosphate (CP), glomerular reclamation, proximal tubule cell, long COVID, arginine-glycine-amidinotransferase (AGAT)

## Abstract

Metabolic causes such as altered bioenergetics and amino acid metabolism may play a major role in Long COVID. Renal-metabolic regulation is an integral part of these pathways but has not been systematically or routinely investigated in Long COVID. Here we discuss the biochemistry of renal tubular injury as it may contribute to Long COVID symptoms. We propose three potential mechanisms that could be involved in Long COVID namely creatine phosphate metabolism, un-reclaimed glomerular filtrate and COVID specific proximal tubule cells (PTC) injury-a tryptophan paradigm. This approach is intended to allow for improved diagnostics and therapy for the long-haul sufferers.

## 1. Introduction

The USA has suffered over 1 million deaths from COVID-19. The Centers for Disease Control (CDC) has confirmed approximately 95 million persons with a positive test for COVID-19 (1). Multiple scenarios predict this number is grossly underestimated (2). Our focus is on the additional tragedy of 1 to 12% (3) of the hundreds of millions infected world-wide may experience a protracted course of illness, variably called Long or Long-haul COVID or Post-Acute COVID Sequelae (PACS) (4, 5).

No consensus definition of this syndrome has been promulgated. European authorities (6) and in the USA, the American Academy of Physical Medicine and Rehabilitation (7) and the Kaiser Permanente Mid-Atlantic group (8) have all offered guidance regarding diagnosis and/or management of Long COVID. A study from the Netherlands has attempted to refine the definition by correcting for pre-existing symptoms and using age and gender-based controls (3). Long COVID may be indistinguishable from the prolonged recovery that occurs after acute severe disease as in the case of patients requiring mechanical or pharmacological life support. But a significant proportion of Long COVID sufferers had milder initial infections (9). Many did not require hospitalization (5). Long COVID is expressed in over 200 symptoms involving ten physiologic systems (9, 10), but among these fatigue (with a prevalence of 98%) and cognitive impairment (often termed Brain Fog with a prevalence of 85%) are the cause of significant debility and decreased quality of life (9, 10, 11). The focus of this communication is the biochemical pathogenesis of muscle fatigue and Brain Fog and the involvement of renal tubular physiology. Thirty years ago, scientists at Oxford University and the Karolinska Institute articulated multiple metabolic causes of muscle fatigue and brain impairment (12). Of these metabolic causes, altered bioenergetics and amino acid metabolism play a major role. Thus, renal-metabolic regulation is an integral part but has not been systematically or routinely investigated in Long COVID. Here we discuss the biochemistry of renal tubular injury as it may contribute to Long COVID symptoms. This approach is intended to allow improved diagnostics and therapy for the Long-haul sufferers.

Renal dysfunction is a well-established cause of metabolic disturbances and toxin accumulations that impair muscle and brain function (13, 14). Renal disorders of sodium, potassium, magnesium, calcium, phosphate, and pH are familiar causes of metabolic myopathies and encephalopathies and therefore will not be further discussed here (15). We emphasize less heralded pathways that are potentially disturbed after COVID kidney injury.

The incidence of acute kidney injury (AKI) in hospitalized patients with COVID varies during the time course of the disease and according to demographic factors. Teixiera et al. report AKI incidence in hospitalized COVID patients in the USA at 33–57% with about a third of these requiring renal replacement therapy (16). Acute kidney injury in COVID is often multifactorial with the preponderance of morphologic injury being in the renal tubules. Recent evidence from kidney biopsies and post-mortems has disclosed significant injury to the proximal tubular cells (PTC) (17). The injury was demonstrated by abnormal urinalysis and biochemical evidence in the urine of PTC dysfunction including phosphaturia, uricosuria, aminoaciduria, and low molecular weight proteinuria (17). Biomarkers and detailed light and electron microscopy disclosed marked renal tubular cell damage. Immune staining revealed marked PTC decrements in Megalin and URAT-1 transporter. Direct severe acute respiratory syndrome coronavirus viral invasion was confirmed by transmission electron microscopic evidence of viral particles but with the caveat that the observed particles may be artifactual. However, other investigators have found viral particles by electron microscopy in the tubules and podocytes and confirmed the presence of SARS-CoV-2 nuclear protein antigen. The pathology illustrated the most extreme, in that most specimens were postmortem (17).

A cogent argument for the role of direct viral infection by SARS-CoV-2 in PTC injury has been presented by Soleimani (18). There follows a meticulous discussion of the complex physiologic derangement of the infected PTC (18). Renal PTC injury would be expected during the viremic stage of COVID given the high density of the SARS-CoV-2 receptor, ACE2, on the PTC membrane (17). Consequent perturbations of renal tubular function are discussed under the three sections on the proposed mechanisms generating Long COVID symptoms.

Only a minority of Long COVID patients present with a history of AKI. However, PTC dysfunction that is not associated with diminished urine output or rising serum creatinine may be unrecognized. Our search was unable to find renal tubular function data on unselected hospitalized COVID patients. In the study of Werion et al. normoglycemic glycosuria was not found, in contrast to the marked defects in reabsorption of urate, phosphate and amino acids. Little information was offered regarding proximal renal tubular acidosis. These observations are critical, since the clinician who reviews the urine dipstick values will be unlikely to search for proximal tubular damage in the absence of unexpectedly alkaline urine or glycosuria. If PTC dysfunction is diagnosed and hypouricemia/uricosuria is present, the morbid risk for the COVID patient is increased, according to this one source (17). We hypothesize plausible mechanisms that may explain, at least in part, the Long COVID symptoms of “brain fog” and muscle fatigue.

## 2. Mechanism I: Bioenergetic impairment-creatine phosphate and GAA deficiency syndromes

**Figure 1:**
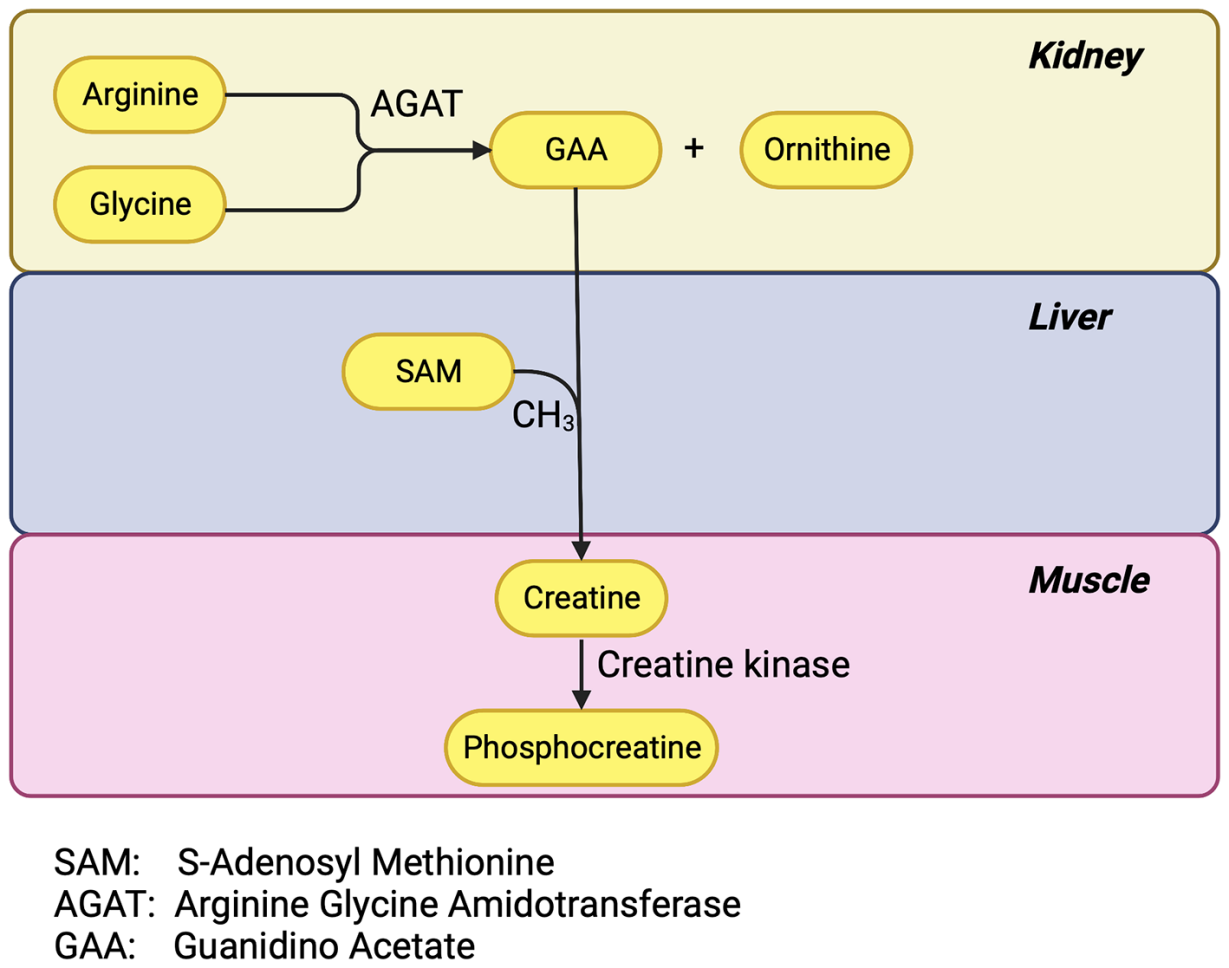
Phosphocreatine pathway.

Generalized PTC injury impairs phosphocreatine synthesis. The PTC has a high concentration of the enzyme arginine-glycine-amidinotransferase (AGAT) (19). AGAT is the first and rate-limiting enzymatic step in phosphocreatine synthesis. The renal tubule uses phosphocreatine as an energy source during periods of high demand but 95% of the body’s creatine is in skeletal and cardiac muscle. Most of the remaining 5% is in the brain and red blood cells (20). Creatine phosphate is a major energy source for skeletal muscle (20). Two-thirds of the intracellular creatine is in the form of phosphocreatine which, *via* the catalysis of creatine kinase, can replete consumed ATP faster than by glycolysis or oxidative phosphorylation (Figure 1).

The AGAT reaction yields guanidinoacetate (GAA) and ornithine. The renal tubule exports much of the GAA to the liver for the final step for creatine synthesis and the liver supplies much of the rest of the body including muscle and brain (21). Inherited defects in AGAT cause muscle fatigue and learning and language difficulties (22). Investigators have postulated that GAA deficiency as well may be an under recognized depletion syndrome (19).

Symptomatic deficiency would be most prominent in those with anorexia and malnutrition, on a vegetarian diet, and those with accelerated renal or enteric losses of creatine and in those with diminished functional PTC mass. Malnutrition risk as a negative prognostic factor in COVID-19 patients is recognized (23). Malnutrition is one of the main causes of immunodeficiency, affecting both the innate and the adaptive immune response (24), exposing individuals to an elevated risk of infection (25) and a lower capacity to inhibit viral proliferation. Malnutritional risk is also a negative prognostic factor for mortality, hospital length of stay and clinical status at discharge in patients with COVID-19 (23, 26).

Adipose tissue-induced inflammation in obesity leads to metabolic disturbances that could lead to complications such as dyslipidemia, hypertension, diabetes and cardiovascular disease. ACE2 on adipocytes exerts systemic effects on the cardiovascular system (27). Interactions among sex, adipocyte ACE2, and complications of obesity, have been reported (28). Leptin is one of the most important adipokines driving these pro-inflammatory effects and higher leptin is associated with increased Angiotensin II levels as well as decreased ACE2 expression and activity (29). Thus, the impairment of this one PTC reaction could contribute to Long-haul symptomatology.

## 3. Mechanism II: Un-reclaimed glomerular filtrate

**Figure 2:**
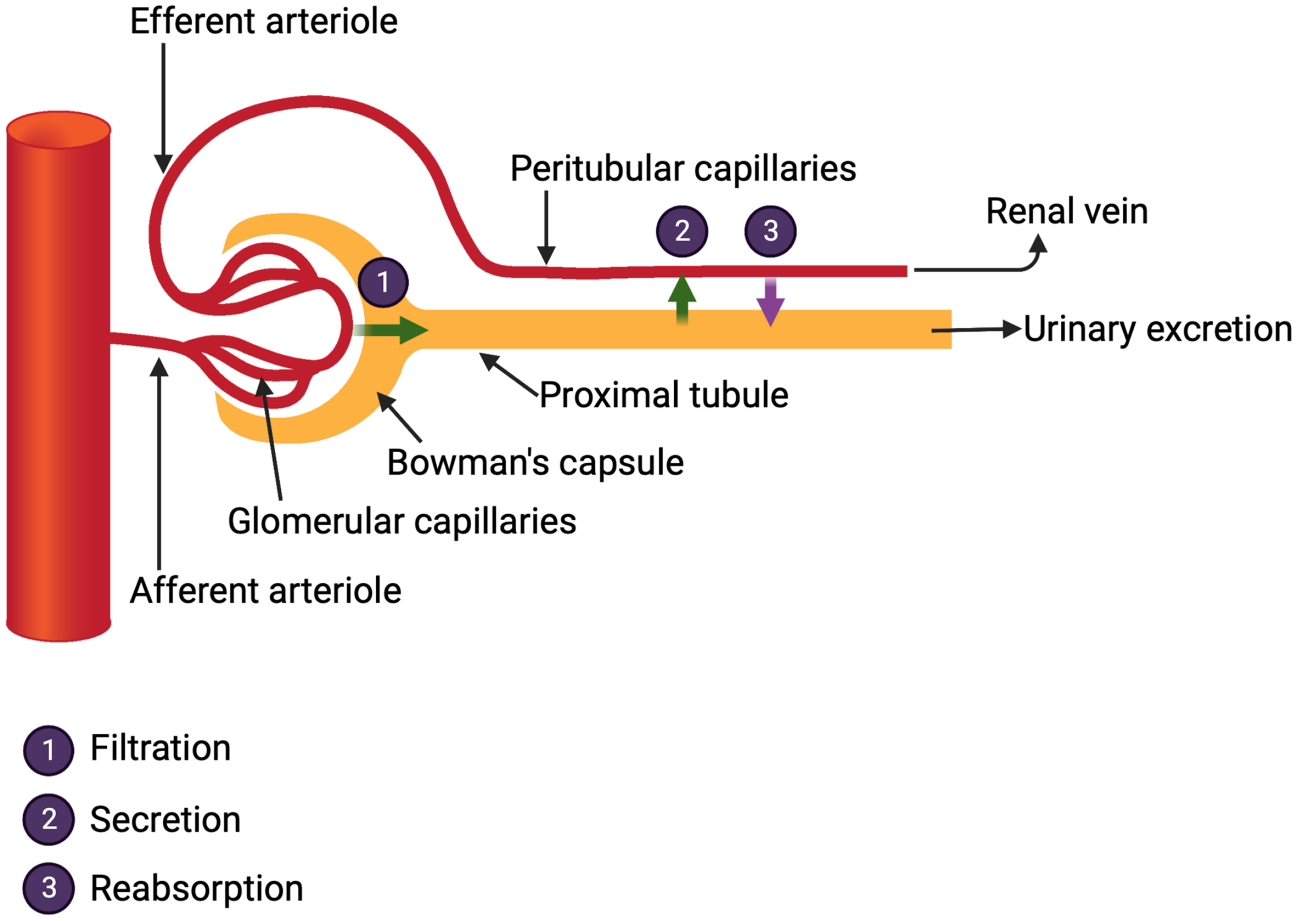
Glomerular functions.

The kidneys receive one fifth of the cardiac output and from this the glomeruli generate 176 liters of filtrate per day in a 70 kg man. This filtrate is rich in molecules essential to the body including water, glucose, electrolytes, minerals, bicarbonate, phosphate, amino acids, albumin and small proteins, e.g., transthyretin (pre-albumin) that carry critical vitamins. It is the life-sustaining and gargantuan responsibility of the renal tubular cells to reabsorb and return to the body about 99% of these substances. The proximal tubule does the heavy lifting in this regard. Curiously, in clinical medicine, even when managing acute kidney injury, few direct measures of this reabsorptive function are routinely ordered. Kidney function in the hospital is assessed by urine output and measurements of plasma urea and creatinine levels. These tests reflect only the renal excretory function which, albeit critical, offers little information of the myriad other kidney functions (Figure 2).

The reclamation of glomerular filtrate is critical and is mediated by multiple membrane transporters for small molecules and by efficient endocytosis for proteins that enter the filtrate.

During COVID infection with AKI, dysfunction of the array of transporter molecules could result in electrolyte and mineral abnormalities that could contribute to Long COVID symptomatology.

The usurpation of the endocytotic pathways in the renal proximal tubule has been meticulously described (18, 30). The latter reference includes details on various mechanisms SARS-CoV-2 translocation in intestinal and other cells as well. COVID AKI is frequently associated with albuminuria. This could result from direct glomerular injury and/or failure of proximal tubular endocytosis. The glomerular podocyte expresses ACE2, so it is presumed susceptible to SARS-CoV-2 attack. However, podocyte injury is seen in an array of glomerulopathies including diabetes, a condition with increased risk for COVID infection. Silva-Aguiar has reported that the SARS-CoV-2 spike protein inhibits Megalin-mediated albumin endocytosis (31). Thus, PTC injury could account for a component of the albuminuria.

In the kidney, ACE2 is strongly expressed in the brush border of proximal tubular cells and some in parietal epithelial cells and podocytes, whereas ACE2 staining is weak or negative in glomerular endothelial cells and mesangial cells. SARS-CoV-2 infections seem to be more frequently associated with AKI compared with SARS-CoV-1. The increased binding affinity of SARS-CoV-2 to ACE2 may explain this phenomenon, as it would allow for greater renal infectivity. More detailed mechanism of SARS-CoV-2 effects on various components of the renin angiotensin axis is described elsewhere and is beyond the scope of this hypothesis.

Viral invasion and/or secondary inflammation may injure the PTC’s endocytotic pathway (18). We know from loss of function mutations affecting renal endocytosis that pathology results. A notable example is the mutations in Megalin and Cubilin which form the master effector of renal endocytosis. Their disruption causes low molecular weight proteinuria, including protein carriers of thyroid hormones, vitamin B12 and vitamins A and D. These vitamins are normally stored by the body so symptomatic depletion would not be immediate, but if persistent loss occurs, symptoms will appear months after the acute COVID and thus manifest during the Long COVID phase. However, an earlier onset of symptoms should be expected in those with prior nutritional compromise. Deficiencies of the fat-soluble vitamins A and D give rise to cutaneous, visual and immune symptoms as well as disruption of calcium homeostasis. Deficiency of vitamin B12 causes changes in sensation, proprioception and balance that can lead to ataxia, cognitive challenges and severe anemia (32). In fact, in the inherited defect of Cubilin, despite chronic proteinuria, the long-term kidney excretory function is preserved but those affected have significant risk of neurologic and hematologic sequelae, possibly related to those seen in Long COVID.

## 4. Mechanism III: COVID specific PTC injury-a tryptophan paradigm

**Figure 3:**
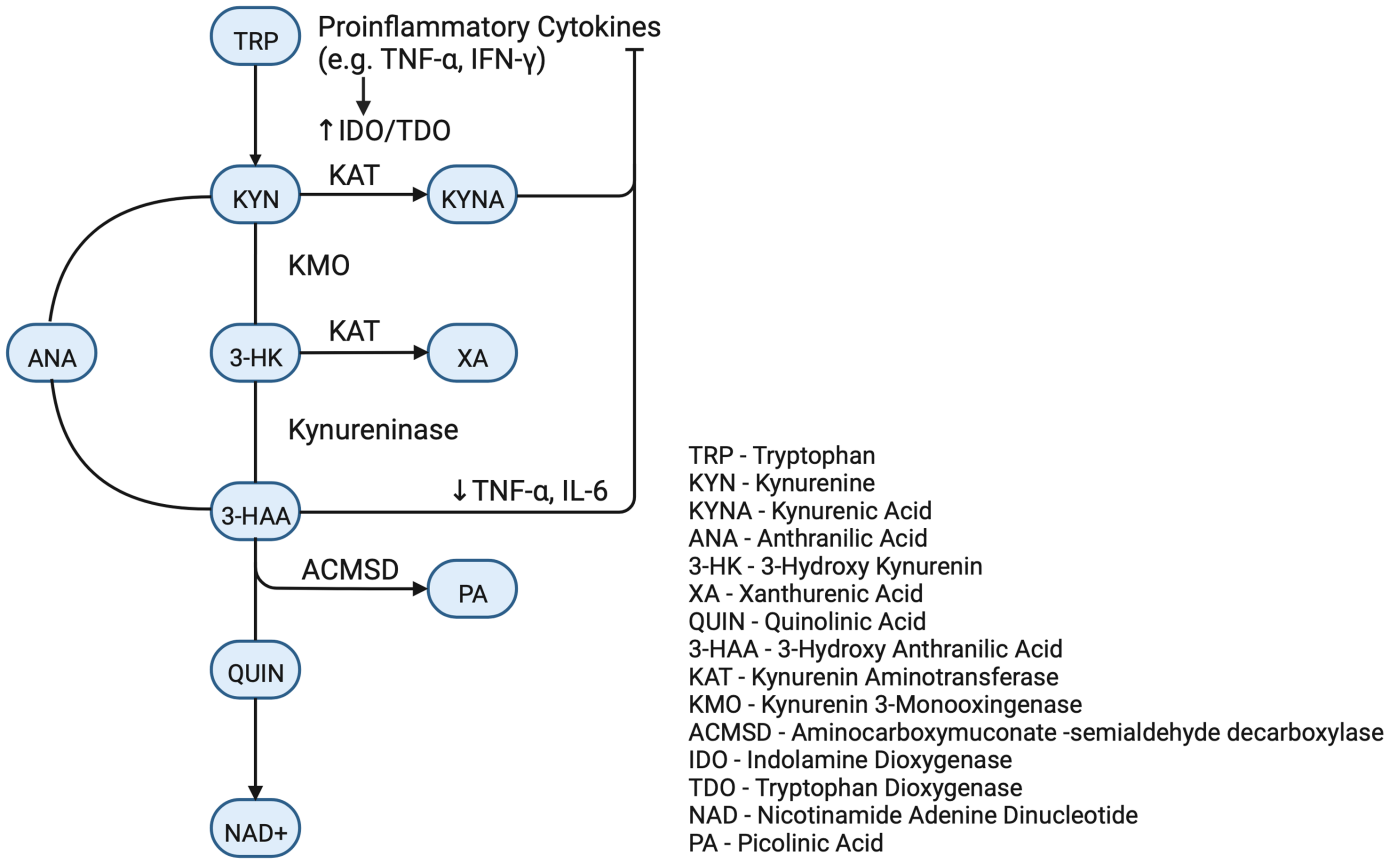
Tryptophan/kynurenine pathway.

Direct SARS-CoV-2 viral infection of the renal parenchyma has been documented (17, 18, 33). The viral spike protein binds to the copious ACE2 receptors of the proximal tubule. This is followed by proteolytic cleavage by one of several enzymes. The function of the proteases is to facilitate the viral endocytotic odyssey to the lysosome and ultimate release of RNA to be replicated by the host’s machinery (Figure 3).

SARS-CoV-2 infection of the renal PTC not only abrogates the function of ACE2, but also alters the function of the ACE2 homolog, Collectrin (34). Collectrin, although lacking the catalytic function in ACE2, apparently has chaperoning function for other membrane proteins including B(0)AT1, the Hartnup transporter of neutral amino acids, in both the kidney and intestine. The intestine is the organ richest in ACE2 receptors and COVID can result in altered B(0)AT1 transport in the gut as well. In the gut, B(0)AT1 membrane trafficking is independent of Collectrin and the chaperone function is accomplished directly by ACE2 (35). The disruption of renal and intestinal transport of neutral amino acids is a known cause of medical syndromes, with Hartnup’s disease being the genetic prototype (36). An instructive example of neutral amino acid deficiency is that of defective Tryptophan transport. This emphasis is not to deny the involvement of other neutral amino acids, for example branch chain amino acids, a dearth of which could contribute to decreased muscle performance.

Depletion of Tryptophan disrupts critical biochemical pathways. Tryptophan has multiple metabolic fates. Best known of these is the pathway to Serotonin utilized by neural tissue and gut and the further metabolism to produce Melatonin *via* the “night reaction” in the pineal gland (37). Low Serotonin levels are associated with multiple neuro-psychiatric afflictions (38) and loss of Melatonin is implicated in dysregulation of sleep (39) as is a catabolite and photo-product of Tryptophan abbreviated FICZ [6-formylindolo(3,2-b)carbazole], which informs the neural systems that control circadian rhythms (40). Derangement of these processes could explain some of the neuro-cognitive symptoms evident in Long COVID.

Although the Serotonin pathway is most widely recognized, the vast majority of Tryptophan is metabolized *via* the Kynurenine Pathway (KP) (41). The initial biochemical step in the committed KP is catalyzed by three dioxygenase enzymes, IDO-1, IDO-2, and TDO. These enzymes are even accelerated during acute disease as IDO-1 and IDO-2 are stimulated by inflammatory cytokines (IL-1, IL-6, and TNF-alpha) and TDO is stimulated by the stress hormone cortisol and, perhaps, the COVID therapeutic, Dexamethasone (41). Kynurenine is further metabolized to neuro-modulatory compounds and to nicotinamide adenine dinucleotide (NAD+). NAD+ is a ubiquitous cofactor in cellular bioenergetics and precursor to NADP, the phosphorylated derivative of NAD+, which is essential in biosynthetic and redox reactions. Deprivation of NAD+ leads to pellagra which manifests as photosensitive dermatitis, diarrhea, and cognitive dysfunction.

Kynurenine (KYN) and Kynurenic Acid (KynA) are important metabolites with several critical realms of function. Kynurenine is active in the brain as evidenced by specific brain transporters and that 60–80% of the brain Kynurenine is imported from other tissues (41). The impact of altered KP on COVID associated cognitive defects is speculative. The downstream metabolites of Tryptophan (called TRY CATS) are involved in a multiplicity of cerebral functions. The enzyme kynurenine aminotransferase (KAT) converts KYN to KynA and both are known modulators of neuroprotection, attenuators of glutamate stimulation and down regulators of the alpha7 nicotinic receptor component of the parasympathetic nervous system. KynA also attenuates aspects of the sympathetic branch of the autonomic nervous and regulates astrocytes by activation of the GPR35 membrane receptor. This activation also reduces inflammation (41). As inflammation may be a significant factor in brain fog and muscle fatigue, it must be noted that Kynurenine and KynA are primary ligands for the Aryl hydrocarbon receptor (AhR) (42), an ancient suppressor of the innate immune system (41, 43). Absence of effective innate immune suppression is a putative contributor to cytokine storm which is a major pathogenic factor in severe acute COVID. AhR, which is widely expressed in the central nervous system, may augment neuroprotection *via* its recently described activity as a transcription factor for Neprilysin, an important endopeptidase involved in the degradation of amyloid (44). KynA, as an AhR agonist, has been shown to increase Neprilysin activity in human neuroblastoma cells (44).

Assessing the ultimate effect of altered Tryptophan and TRY CATS on mental function and muscle fatigue is perilous. The panoply of TRY CATS, their membrane receptors and transporters and associated interactants confounds any simplistic paradigm for predicting clinical syndromes. This is exemplified in the analysis of muscle fatigue. Investigators delineate two modes of muscle fatigue: a “peripheral” form that arises from intrinsic myocyte bioenergetics and a “central” form due to the neurology of the perception of effort (45). Within this categorization, TRY CATS may have opposing influences. Tryptophan and metabolites may be beneficial to the muscle cell, while in the CNS excess conversion of Tryptophan to Serotonin is a putative cause of central fatigue (45). Although we have emphasized mechanisms that limit the amount of Tryptophan, during muscular exercise, the displacement of Tryptophan from its protein binding sites by mobilized free fatty acids would cause an abrupt increase in the free level (45) which promotes Tryptophan entry into the myocyte.

More detail on the Tryptophan/KP in acute COVID is provided from the University of Colorado COVIDome Project (46) and in collaboration with Columbia University and the University of Virginia (47). This is a multi-omics analysis of blood from hospitalized patients with severe COVID with comparison made to convalescent donors and COVID negative hospitalized patients. The data was additionally stratified according to IL-6 level, as IL-6 is a marker of cytokine release. Tryptophan metabolism was the most affected pathway and changes were greatest in patients with the highest Il-6 concentrations. Tryptophan mono-oxygenation pathway was significantly depressed with low levels of Tryptophan, Serotonin, Indoleacetates and Indolepyruvates. The KP pathway was hyper-activated as is consistent with the known stimulus of IDO and TDO by inflammatory and stress mediators. The COVIDome reported preliminary sequential data on two patients hospitalized with severe COVID. One patient survived and one succumbed to the disease. In the survivor there was a robust Kynurenine production which was temporally associated with Il-6 declining while in the deceased patient there was a very attenuated production of Kynurenine and sustained elevation of IL-6 and other inflammatory markers. Although this pattern must be confirmed in many additional patients, it is consistent with the proposition that Kynurenine or KynA induced activation of the Aryl hydrocarbon receptor is a component of the suppression of the hyperimmune response.

As previously mentioned, attenuation of the small intestinal B(0)AT1 transporter also can occur in COVID infections as the intestine has one of the highest concentrations of ACE2. Researchers have documented positive stool PCR for COVID virus in Long COVID sufferers (48, 49). A disruption of dietary neutral amino acid absorption would exacerbate the deficiencies caused by renal tubulopathy. Malabsorbed Tryptophan enters the colon where the colonic microbes degrade Tryptophan to toxic products. The best studied of these has been indoxyl sulfate, a proposed uremic toxin, with the ability to displace hormones, medications and fatty acids from their binding sites on albumin. This fact could disrupt intermediary metabolism and enhance drug toxicity (50). Myriad other degradation products from other unabsorbed amino acids are likely as well. In analogous gut-derived toxidromes (e.g., auto-brewery syndrome and d-lactic acidosis), patients suffer weakness, cognitive deficits, and prolonged illness (51).

We have utilized altered Tryptophan metabolism as a model for the pathophysiology that could occur with COVID induced abrogation of neutral amino acid transport. Parallel disturbances of physiology may accrue from the loss of the other neutral amino acids; the potential physiologic perturbations are beyond the scope this paper.

## 5. Discussion

The above proposed pathogenesis invokes a narrow focus on less widely emphasized biochemistry. This nephro-centric theory/hypothesis is in no way intended to account for the majority of Long COVID symptoms. This is not an attempt to diminish the contributions of other organ systems. In fact, it is our fervent hope that as pathogenetic paradigms continue to emerge from all COVID affected systems, a more complete understanding of Long COVID will eventuate. We have proposed three potential mechanisms by which some metabolic disturbances caused by COVID could translate into several manifestation of the ambiguously defined Long COVID syndrome.

## Data availability statement

The original contributions presented in the study are included in the article/supplementary material, further inquiries can be directed to the corresponding authors.

## Author contributions

RG and DB-O contributed to conception and writing the manuscript and wrote the first draft of the manuscript. PC critically reviewed the manuscript. All authors contributed to the article and approved the submitted version.

## Conflict of interest

The authors declare that the research was conducted in the absence of any commercial or financial relationships that could be construed as a potential conflict of interest.

## Publisher’s note

All claims expressed in this article are solely those of the authors and do not necessarily represent those of their affiliated organizations, or those of the publisher, the editors and the reviewers. Any product that may be evaluated in this article, or claim that may be made by its manufacturer, is not guaranteed or endorsed by the publisher.
